# Serum Calcification Propensity and Calcification of the Abdominal Aorta in Patients With Primary Aldosteronism

**DOI:** 10.3389/fcvm.2022.771096

**Published:** 2022-01-24

**Authors:** Marta Kantauskaite, Katharina Bolten, Matthias Boschheidgen, Claudia Schmidt, Thilo Kolb, Kai Uwe Eckardt, Andreas Pasch, Lars Schimmöller, Lars C. Rump, Jakob Voelkl, Johannes Stegbauer

**Affiliations:** ^1^Department of Nephrology, Medical Faculty, University Hospital Düsseldorf, Heinrich-Heine-University Düsseldorf, Düsseldorf, Germany; ^2^Department of Diagnostic and Interventional Radiology, Medical Faculty, University Hospital Düsseldorf, Heinrich-Heine-University Düsseldorf, Düsseldorf, Germany; ^3^Department of Nephrology and Medical Intensive Care, Charité-Universitätsmedizin Berlin, Berlin, Germany; ^4^Institute for Physiology and Pathophysiology, Johannes Kepler University Linz, Linz, Austria; ^5^Calciscon AG, Biel, Switzerland; ^6^German Centre for Cardiovascular Research (DZHK), Partner Site Berlin, Berlin, Germany

**Keywords:** aldosterone, primary aldosteronism, hypertension, vascular calcification, serum calcification propensity

## Abstract

Patients with primary aldosteronism (PA) are more susceptible to cardiovascular disease and mortality than patients with primary hypertension. This is mostly attributed to excess production of aldosterone and its effects on the development of vascular injury. A novel functional test (T_50_) measures serum calcification propensity. Lower T_50_-values predict higher cardiovascular risk. We investigated serum calcification propensity and vascular calcification in PA and resistant hypertension (RH). T_50_ measurement was performed in patients with PA (*n* = 66) and RH (*n* = 28) at baseline and after 403 (279–640) and 389 (277–527) days of treatment. No significant differences in T_50_-values were observed between the groups (371 ± 65 and 382 ± 44 min, in PA and RH group, respectively, *p* > 0.05). However, higher aldosterone-to-renin ratios were associated with lower T_50_-values in PA-patients (*r* −0.282, *p* < 0.05). Furthermore, lower T_50_-values were associated with increased abdominal aortic calcification measured by Agatston score in PA (*r* −0.534, *p* < 0.05). In both, PA and RH, higher atherosclerotic cardiovascular disease (ACSVD) scores (*r* −0.403, *p* < 0.05) and lower HDL (*r* 0.469, *p* < 0.05) was related to lower T_50_-values in a linear regression model. Adrenalectomy or medical treatment did not increase T_50_-values. In comparison to patients with stable T_50_-values, PA patients with a decrease in T_50_ after intervention had higher serum calcium concentrations at baseline (2.24 ± 0.11 vs. 2.37 ± 0.10 mmol/l, *p* < 0.05). This decline of T_50_-values at follow-up was also associated with a decrease in serum magnesium (−0.03 ± 0.03 mmol/l, *p* < 0.05) and an increase in phosphate concentrations (0.11 ± 0.11 mmol/l, *p* < 0.05). Resistant hypertension patients with a decrease in T_50_-values at follow-up had a significantly lower eGFR at baseline. In summary, these data demonstrate an association between a high aldosterone-to-renin ratio and low T_50_-values in PA. Moreover, lower T_50_-values are associated with higher ACSVD scores and more pronounced vascular calcification in PA. Thus, serum calcification propensity may be a novel modifiable risk factor in PA.

## Introduction

Primary aldosteronism (PA), one of the most common causes of secondary hypertension, is the consequence of the excessive autonomous production of aldosterone either by an aldosterone producing adenoma (APA) or by bilateral hyperplasia (1). Increased mineralocorticoid-receptor activation is associated with hypertension and cardiovascular aging (2, 3). Patients with PA develop and die more often from cardiovascular diseases than patients with primary hypertension (4). A contributing factor could be aldosterone-induced vasculopathy and vascular calcification, promoting the development of arterial stiffness (5–9). Vascular calcification has been discussed as an important cardiovascular risk factor, as the presence of vascular calcification was associated with an up to four times increased cardiovascular mortality in a meta-analysis (10).

Vascular calcification is a multifactorial and regulated process, augmented by active mechanisms involving vascular smooth muscle cells (11). These cells may undergo an osteogenic reprogramming upon exposure to calcification-stimuli and subsequently promote an extracellular environment favoring mineralization (11). Elevated aldosterone concentrations augment osteogenic reprogramming and calcification of vascular smooth muscle cells (3, 12–14). Furthermore, excessive mineralocorticoid receptor (MR) activation has been associated with endothelial dysfunction, activation of myeloid cells, and vascular inflammation as well as other vascular alterations, which may promote vascular calcification (13, 15–17). Accordingly, recent studies have shown that patients with PA have more pronounced vascular calcification than age matched controls with essential hypertension (EH) (18, 19). Ectopic vascular calcification is closely associated with dysregulated phosphate homeostasis. Phosphate can precipitate with calcium, a process prevented by systemic and local calcification inhibitors (20). An important role in the prevention of ectopic calcification is attributed to formation of calciprotein particles (CPP) to clear calcium-phosphate ion clusters from blood and tissues (21). The insufficient clearance and maturation of spontaneously formed primary CPPs toward a secondary larger form with crystalline core has been termed as “mineral stress.” Secondary CPPs induce osteogenic reprogramming and calcification of vascular smooth muscle cells, a process which is suspected as key culprit in the promotion of vascular calcification by phosphate (21). The performance of this functional system can be assessed in serum samples by the T_50_ calcification propensity test (22). The T_50_ test determines the half maximal transformation time (T_50_) of primary CPPs to the secondary insoluble CPPs, where lower T_50_ is associated with increased serum calcification propensity (22). Thus, the presence of anti-calcific factors prolongs this time, while reduced “anti-calcific defense” shortens this time. Factors modifying this assay are e.g., calcium, phosphate, magnesium, and anti-calcific proteins such as Fetuin-A (22), but further currently unknown determinants may be involved in the test results. As an overall functional assay, the results could be interpreted as how prone an individual patient serum is toward calcification. The importance of serum calcification propensity was confirmed in general (23), pre-dialysis (24), dialysis (25), kidney transplant populations (26) where it outperformed the traditional risk factors.

Since elevated aldosterone concentrations promote phosphate-induced vascular calcification (12), we investigated whether PA patients are prone to worse serum calcification propensity. Therefore, we measured serum calcification propensity (T_50_) in patients with PA. To accurately elucidate the role of aldosterone, we compared PA patients to blood pressure matched patients with resistant hypertension (RH) with no history of PA. Moreover, by measuring T_50_ at the diagnosis and 1 year after treatment initiation we were able to present intra-individual changes in this population.

## Methods

### Study Population

A total of 66 PA patients and 28 age, gender, blood pressure matched patients with RH were recruited for this observational prospective study. The study was approved by the local ethics committee (Study numbers: 3848 and 3919) and carried out in accordance with the Declaration of Helsinki. All patients provided written informed consent to participate in the study.

All patients enrolled into the study underwent examinations according to previously published guidelines in order to exclude or confirm secondary causes of hypertension (27). Patients with abnormal aldosterone renin ratio (ARR) after discontinuation of interacting antihypertensive drugs were further investigated and received an intravenous salt loading test to confirm the diagnosis PA. To determine whether unilateral or bilateral adrenal gland involvement is responsible for the excessive aldosterone production, all patients underwent selective adrenal vein sampling and imaging of the adrenal glands either by CT-scan or MRI. Patients with unilateral masses in the imaging and unilateral autonomous hormone production were advised to undergo adrenalectomy. In such cases, the diagnosis of an APA was confirmed histologically. Patients who showed lateralization in the selective adrenal vein sampling but were not operated, were referred to as unilateral hyperplasia (ULH) and treated with MRA. Patients with bilateral adrenal hyperplasia were treated with MRA. According to the ESH/ESC guidelines (28), patients were defined having RH if under the prescription of three different antihypertensive drugs including a diuretic hypertensive blood pressure values were observed. In addition, these patients received examinations in order to rule out secondary causes of hypertension. Of note, due to the often asymptomatic course of hypertension and the prolonged diagnosis of PA and RH, we do not have the exact date when our patients developed PA or RH.

Demographic data such as age, gender, body weight, the etiology of PA, and leading comorbidities, were collected at the time of the study entry from medical records. Blood samples were drawn at study entry and at the follow up visit 12 months after treatment initiation which in the case of PA was adrenalectomy or drug therapy with MR blocker. Resistant hypertension patients received adjustments in antihypertensive treatment. At both visits patients received 24 h blood pressure monitoring. The measurements were performed using Mobilograph PWA system (IEM GmbH, Stolberg, Germany). During the day blood pressure was measured every 15 min whereas at night every 30 min. The size of the cuff was chosen individually. Laboratory parameters such aldosterone, renin, ARR, electrolytes, creatinine, albumin, C-reactive protein were assessed using standard methods.

### Cardiovascular Risk Score

Risk of major cardiovascular complications such as heart disease and stroke was assessed using atherosclerotic cardiovascular disease (ACSVD) score which was proposed by the American College of Cardiology and the American Heart Association (ACC/AHA) in 2013 (29). Calculator includes parameters as age, gender, race, blood pressure values and treatment situation, concentrations of total cholesterol, and high density lipoprotein (HDL), smoking status, history of diabetes. It is important to note, that ACSVD score is validated for 40–79 years old patients without a history of heart attack or stroke. The risk is given as percentages. Values between 5 and 7.5% describe borderline risk, 7.5–20% intermediate risk whereas values above 20% are associated with high risk for major cardiovascular events. For further analysis patients were divided into two groups—the ones with low risk and the ones with ACSVD score above 7.5% representing intermediate and high risk pattern.

### Calcification Propensity Measurement (T_50_)

Fasting blood samples were drawn from a peripheral vein in vacuum tubes and immediately centrifuged at 3,000 rpm for 15 min at ambient temperature. The extracted serum was stored in aliquots and was frozen at −80°C until further use. Calcification propensity score was determined as the half maximal transformation time from primary to secondary CPP—T_50_ at the Calciscon Laboratory in Switzerland (22), which was blinded for the patients.

### Measurement of Vascular Calcification in Computer Tomography

At the study entry patients diagnosed with PA underwent either an unenhanced abdominal CT scan or MRI in order to confirm adrenal adenoma or hyperplasia. Available CT scans were assessed using Syngo.via Calcium Scoring software (Siemens Medical Solutions, USA) which is originally used to evaluate the extent of coronary calcification (30). Similar to the evaluation of coronary arteries, calcified areas with density above 130 HU (Hounsfield units) of aorta from diaphragm to bifurcation were marked by two independent observers with experience in computed tomography and calcium scoring, which enabled the assessment of calcification volume, equivalent mass, peak density, and Agatston score as described previously (31). Agatston score is a software tool based on the weighted density score given to the highest attenuation value multiplied by the calcified area.

### Statistical Analysis

Statistical analysis was performed using SPSS version 23 (SPSS Inc., Chicago, USA) and Graph Prism 5.3 (GraphPad Software, San Diego, USA). Continuous data are expressed as mean ± standard deviation (SD) or median (interquartile ranges expressed as two numbers, Q1–Q3, respectively) whereas categorical data are expressed as number (percentage). Shapiro-Wilk test was used to test if collected data is normally distributed. The difference among groups was evaluated using chi-square, *t*-test, or Mann Whitney test where appropriate. In order to compare the means between entry visit and follow up, paired *t*-test, or Wilcoxon signed rank test was performed. Linear regression was used for indicating variables associated with T_50_. In some cases because of violated homoscedasticity a weighted least squares (WLS) regression was used. Variables having significant effect on T_50_ in univariate linear regression were included into multivariate analysis. Receiver operating characteristic (ROC) curve and Youden test was used to determine T_50_ cutoff values for moderate to severe abdominal aortic calcification risk. A *p*-value < 0.05 was considered statistically significant.

## Results

### Study Population

Baseline characteristics of patients diagnosed with PA and RH are presented in Table 1. No differences in gender, age, blood pressure, renal function, and cholesterol levels were present between the groups. By definition, patients with PA had significantly higher aldosterone concentration and ARR levels whereas renin production was markedly reduced (*p* < 0.05). Cardiovascular risk assessment using ACSVD score was lower among PA patients (median score 9.6% among PA group and 24.9% among RH group, *p* < 0.05). This effect could be attributed to the fact that beside high blood pressure, these patients did not have any other factors such as diabetes or smoking history accounting for higher cardiovascular risk. Serum mineral parameters appeared well-controlled and within population-based reference values within the studied groups. T_50_-values at study entry among patients with PA were slightly lower compared to ones observed in RH group, 371 ± 65 min and 382 ± 44 min, *p* > 0.05 respectively (Table 1).

**Table 1:** Baseline characteristics of study groups.

	**All patients**	**PA**	**RH**
Male: Female	1.8:1	1.8:1	1.8:1
Age, years	54.6 ± 11.7	53.5 ± 12.4	58.0 ± 11.2
Diagnosis, *n* (%)			
APA/ULH	36 (38.3%)	36 (54.5%)	0 (0.0%)^***^
BLH	26 (27.7%)	26 (39.4%)	0 (0.0%)^***^
Unclassified	4 (4.2%)	4 (6.1%)	0 (0.0%)^***^
RH	28 (29.8%)	0 (0.0%)	28 (100%)^***^
Systolic BP, mmHg	155 ± 19	154 ± 11	156 ± 21
Diastolic BP, mmHg	90 ± 9	88 ± 8	91 ± 10
ACSVD score, %	13.9 (6.6–24.5)	9.6 (4.7–16.8)	24.9 (17.5–29.9)^*^
Aldosterone, ng/l	148.7 (98–223.7)	187.3 (119–241)	99 (42–148) ^***^
Renin, pg/ml	2.5 (1.6–7.5)	1.9 (1.2–2.8)	11 (4.4–59)^***^
ARR	61.1 (22.1–108)	85.3 (52.4–134.4)	4.6 (0.9–22.9)^***^
ARR at follow up	8 (2.2–26.2)	8.7 (2.5–30.3)	4.0 (0.9–15.4)
Creatinine, mg/dl	0.9 (0.8–1.1)	0.9 (0.8–1.1)	0.9 (0.8–1.3)
eGFR, ml/min/1.73 m^2^	80.5 (69.5–95.5)	82 (72.7–95.5)	74.5 (54.5–96.5)
Calcium, mmol/l	2.31 ± 0.1	2.33 ± 0.1	2.30 ± 0.1
Phosphate, mmol/l	0.99 ± 0.2	0.97 ± 0.3	1.01 ± 0.2
Magnesium, mmol/l	0.84 ± 0.1	0.83 ± 0.1	0.86 ± 0.1
Triglyceride, mg/dl	126 (95–184)	127 (96–183)	119 (93–206)
HDL, mg/dl	47 (38–61)	49 (41–61)	45 (37–57)
Albumin, g/dl	4.4 ± 0.3	4.4 ± 0.3	4.4 ± 0.5
CRP, mg/dl	0.3 (0.2–0.6)	0.3 (0.2–0.5)	0.4 (0.3–1.1)^*^
HbA1_C_, %	5.6 (5.2–6.1)	5.4 (5.1–5.8)	5.8 (5.6–6.4)^*^
T_50_, min	372 ± 59	371 ± 65	382 ± 44
T_50_ at follow up, min	359 ± 54	354 ± 52	367 ± 56

*PA, primary aldosteronism; RH, resistant hypertension; APA, aldosterone producing adenoma; ULH, unilateral adrenal hyperplasia; BLH, bilateral adrenal hyperplasia; BP, blood pressure; ACSVD, atherosclerotic cardiovascular disease score; ARR, aldosterone renin ratio; eGFR, estimated glomerular filtration rate; HDL, high density lipoprotein; CRP, C-reactive protein; HbA1C, glycosylated hemoglobin; T_50_, calcification propensity. Dichotomous data are presented as percentages whereas continuous data as means ± SD or median (Q1–Q3)*.

* *Represent significant difference between the groups with p < 0.05,*

*** *p < 0.001 using t-test, chi-square or Mann Whitney test*.

### Influence of Clinical and Biochemical Factors on Calcification Propensity

In order to assess the relationship between T_50_ and various variables, a linear regression model was established (Table 2). Analyzing the whole cohort as a population for difficult-to-treat hypertension, higher ACVSD score (*r* −0.403, *p* < 0.05) and lower HDL (*r* 0.469, *p* < 0.05) were related to lower T_50_-values. Similar associations were observed analyzing study groups separately. Higher cardiovascular risk score was associated with lower T_50_-value in both groups however statistical significance was met only in the PA group (*r* −0.483, *p* < 0.05). In the RH group an inverse negative relationship between phosphate (*r* −0.422, *p* < 0.05) and a positive relationship between HDL (*r* 0.769, *p* < 0.05) concentration and T_50_ was observed.

**Table 2:** Univariate linear regression assessing the relationship between T_50_ measured at the study entry and various variables.

**Variable**	**All patients**		**PA**		**RH**	
	**β**	***p***	**β**	***p***	**β**	***p***
Age	−0.033	0.775	−0.153	0.276	0.192	0.337
Gender	0.053	0.637	0.093	0.506	– 0.054	0.786
Phosphate	−0.221	0.060	−0.186	0.206	−0.422	**0.036**
Calcium	0.024	0.836	0.040	0.779	0.005	0.982
Magnesium	0.111	0.361	0.044	0.778	0.255	0.209
Potassium	−0.178	0.115	−0.248	0.077	– 0.304	0.115
Creatinine	−0.014	0.903	0.035	0.806	−0.230	0.258
eGFR	−0.009	0.936	−0.019	0.890	0.176	0.370
Albumin	0.111	0.359	0.160	0.279	0.048	0.831
HDL	0.469	**<0.001**	0.259	0.075	0.769	**<0.001**
Triglyceride	−0.030	0.796	−0.043	0.764	– 0.146	0.478
CRP	−0.081	0.485	−0.258	0.070	−0.045	0.823
ACSVD	−0.403	**0.004**	−0.483	**0.002**	−0.138	0.685
Aldosterone	−0.079	0.487	−0.133	0.342	0.281	0.155
ARR	−0.103	0.362	−0.282	**0.041**	0.397	**0.040**

*PA, primary aldosteronism; RH, resistant hypertension; eGFR, estimated glomerular filtration rate; HDL, high density lipoprotein; CRP, C-reactive protein; ACSVD, atherosclerotic cardiovascular disease score; ARR, aldosterone renin ration. Significance with p < 0.05. The bold values emphasize variables having significant effect on T_50_*.

At the time of diagnosis, ARR measurements for PA patients were performed after discontinuation of medications interacting with renin-angiotensin-aldosterone system. In PA patients, there was a significant negative relationship between ARR and T_50_ (*r* −0.282, *p* < 0.05) whereas among RH patients an opposite effect was observed (*r* 0.397, *p* < 0.05). As expected after the treatment, PA patients had normal ARR levels which were similar to those observed among RH patients (median 8.7 (2.5–30.3) and 4.0 (0.9–15.4) among PA and RH patients, respectively, *p* > 0.05). At follow up visit, ARR was positively correlated to T_50_ (*r* 0.441, *p* < 0.05).

Factors which showed significant relationship with T_50_ in univariate analysis were further analyzed using multivariate regression model. High phosphate concentration remained related to lower T_50_ when analyzing whole study population (Supplementary Table 1). There was a significant negative relationship between ARR and T_50_ in PA (*r* −0.365, *p* < 0.05). High HDL concentration and high normal ARR were associated with higher T_50_-values among RH patients in multivariate linear regression model (Supplementary Table 1).

### Assessment of Vascular Calcification

**Figure 1:**
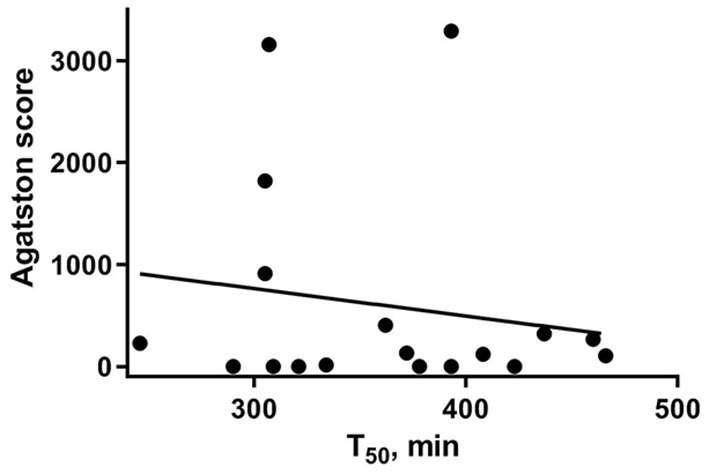
Linear association between calcification propensity measured as T_50_ (X axis) and Agatston score (Y axis), representing the extent of vascular calcification observed in unenhanced CT imaging of abdominal aorta.

Eighteen PA patients with available T_50_-values received abdominal CT scan which enabled the assessment of vascular calcification. There was a negative relationship between T_50_-value and the extent of vascular calcification within abdominal aorta expressed as equivalent mass (*r* −0.557, *p* < 0.05) and as Agatston score (*r* −0.534, *p* < 0.05, as shown in Figure 1). In this patient group, T_50_-values < 338 min had a 69.2% sensitivity and an 87.5% specificity (Youden index 0.567) to identify patients with an Agatston score higher than 100 which indicates moderate or severe abdominal aortic calcification.

### Follow-Up

**Figure 2:**
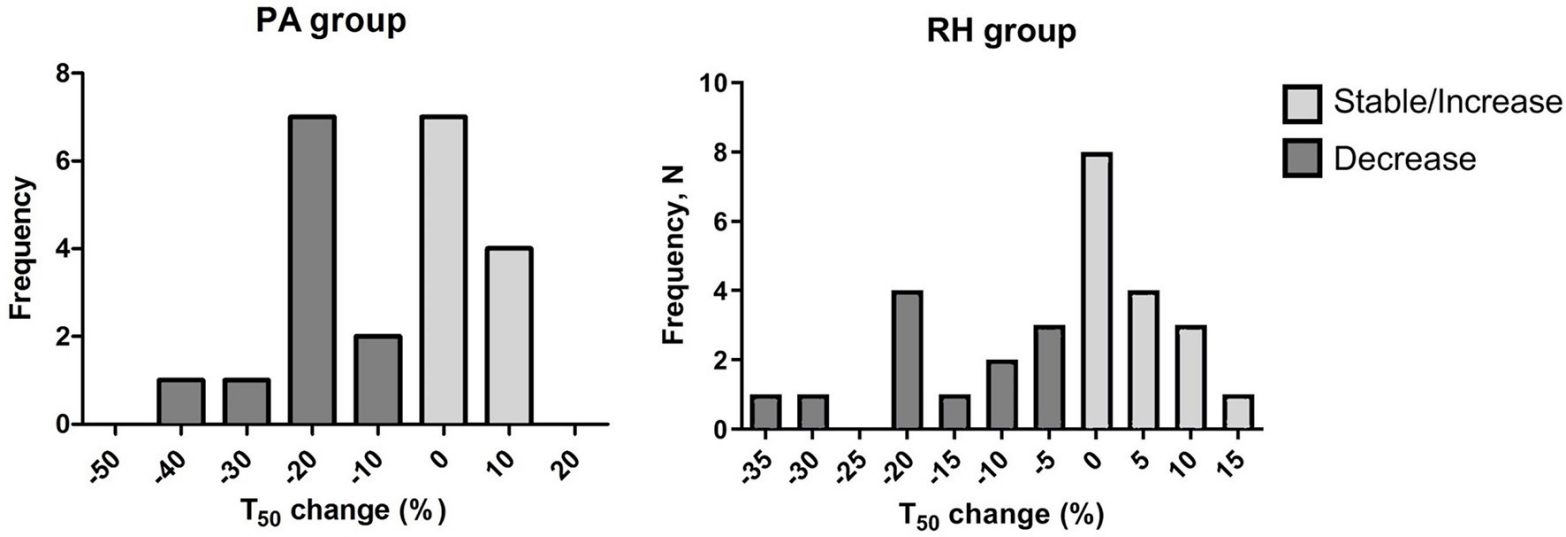
Histogram representing T_50_ changes among patients with PA and RH. Patients with stable or higher T_50_-values over the follow up period were described as stable (gray colored columns) and the ones with a decrease in T_50_ more than 5% compared to the initial value were described as decrease group (dark gray colored columns). Y axis represents the number of patients for each change group.

Primary aldosteronism group was followed-up for a median of 403 (279–640) days whereas RH group for 389 (277–527) days, *p* > 0.05. Following treatment, mean T_50_-values were lower in comparison to baseline values in both study groups (371 ± 65 vs. 354 ± 52 min, *p* < 0.05 among PA patients and 382 ± 44 vs. 367 ± 56 min, *p* > 0.05 among RH group as shown in Supplementary Table 2). To investigate the alteration of T_50_, we divided our patients in two groups according the T_50_ change over the time. Patients with stable or higher T_50_-values over the follow up period were described as stable whereas patients with decrease in T_50_ of more than 5% compared to the initial value were described as decrease group. The distribution of T_50_ change among both groups is demonstrated in Figure 2. Fifty-four percent of patients had stable or increasing T_50_-values following the treatment. Of note, T_50_-values did not differ between PA patients treated either by adrenalectomy or by MR antagonist (data not shown).

As a next step, factors associated with T_50_ change were analyzed. In the PA group, patients with high normal serum calcium concentration at entry tended to have a decrease in T_50_. Mean baseline calcium in T_50_ decrease group was 2.37 ± 0.10 mmol/l whereas patients with stable T_50_-values had lower mean calcium concentration, 2.24 ± 0.11 mmol/l, respectively, *p* < 0.05. Additionally, PA patients in the decreased group showed a significant decline in serum magnesium concentration (−0.03 ± 0.03 mmol/l) and a significant increase in serum phosphate levels (0.11 ± 0.11 mmol/l) over the follow up period.

In the RH group above mentioned variables did not differ between the stable and decrease T_50_ groups. However, in the decrease T_50_ group, significantly lower baseline eGFR values were observed compared to the stable RH group (eGFR 61 (31–86) vs. 92 (77–104) ml/min/1.73m^2^, *p* < 0.05). Of note, a decline in renal function over the follow-up period was associated with a decrease in T_50_, however the statistical significance was not met (Table 3).

**Table 3:** T_50_ changes among the study groups during follow-up.

**Variable**	**PA group**		**RH group**	
	**Stable**	**Decrease**	**Stable**	**Decrease**
Number of patients, *N*	11	11	16	12
Age, years	50.6 ± 8.3	55.5 ± 11.5	56.8 ± 12.9	59.5 ± 12.0
Male:Female	1:1.2	1:0.8	1:0.8	1:0.3
Phosphate, mmol/l	1.02 ± 0.13	0.97 ± 0.25	0.99 ± 0.14	1.08 ± 0.19
Δ Phosphate, mmol/l	−0.04 ± 0.15	0.11 ± 0.11^*^	0.13 ± 0.15	0.22 ± 0.38
Calcium, mmol/l	2.24 ± 0.11	2.37 ± 0.10^*^	2.29 ± 0.11	2.27 ± 0.08
Δ Calcium, mmol/l	0.07 ± 0.07	0.09 ± 0.12	0.04 ± 0.14	0.08 ± 0.13
Magnesium, mmol/l	0.80 ± 0.09	0.83 ± 0.10	0.82 ± 0.08	0.89 ± 0.08
Δ Magnesium, mmol/l	0.03 ± 0.04	−0.03 ± 0.03^**^	0.01 ± 0.07	−0.03 ± 0.09
eGFR, ml/min/1.73 m^2^	89 (75–92)	95 (88–103)	92 (77–104)	61 (31–86)^**^
Δ eGFR, ml/min/1.73 m^2^	−11(−11 to 4)	– 22 (−28 to −6)	−4 (−9 to 0.5)	−4 (−7 to −1.5)
Albumin, g/dl	4.4 ± 0.4	4.6 ± 0.3	4.3 ± 0.4	4.4 ± 0.4
HDL, mg/dl	58 (41–66)	58 (47–69)	51 (40–68)	37 (35–48)
Triglyceride, mg/dl	139 (73–204)	126 (111–145)	116 (91–171)	132 (102–251)
CRP, mg/dl	0.2 (0.1–0.3)	0.4 (0.3–0.8)	0.3 (0.3–0.8)	1.0 (0.3–1.9)
ACSVD score, %	6.6 (3.7–8.9)	13.8 (7.5–16.6)	27 (25.1–29)	24.2 (17.5–25)
Aldosterone, ng/l	186 (119–226)	103 (83.7–222)	76.5 (35.7–152)	127 (46–148)
ARR	93.8 (42.5–119)	53.4 (31–101.1)	4.4 (0.9–25.8)	4.6 (0.4–14.4)

*Patients with stable or higher T_50_-values during the follow-up period were described as stable and the ones with decrease in T_50_ more than 5% compared to the initial value were described as decrease group. PA, primary hyperaldosteronism; RH, resistant hypertension; Δ change between entry and follow up visit; eGFR, estimated glomerular filtration rate; HDL, high density lipoprotein; CRP, C-reactive protein; ACSVD, atherosclerotic cardiovascular disease score; ARR, aldosterone renin ration. Continuous data is presented as means ± SD or median (Q1–Q3)*.

* *p < 0.05,*

** *p < 0.01, difference within the study group (PA or RH) using t-test, chi-square, or Mann-Whitney test*.

## Discussion

The present study explored for the first time the calcification propensity among patients with PA. Here, we show that every ARR increase of 10 was related to 2.8 min reduction in T_50_-values among patients with PA. Secondly, patients with lower T_50_-values had higher ACSVD scores and more pronounced vascular calcification in CT scans. Thirdly, in contrast to our initial hypothesis, normalization of aldosterone concentration either by the surgery or MR blocker therapy did not result in higher T_50_-values.

Serum calcification propensity might be a clinically useful tool to predict and monitor vascular calcification and cardiovascular risk (21). The T_50_ test depends on calcification promoters and inhibitors present in the serum and is associated with cardiovascular mortality (23). Several studies have indicated that T_50_-values decrease in chronic renal failure or diabetes (23, 32). T_50_-values of patients on dialysis are lower than in pre-dialysis patient, and associate with clinical outcomes (24–26). On the other hand, longer duration of CPP maturation determined as higher T_50_-time has been associated with better clinical outcomes, and could be considered an improved calcification propensity with reduced risk for ectopic calcification (23). In the present study, mean T_50_-values among patients with PA were 371 ± 65 min and among RH patients 382 ± 44 min, respectively (23). The current investigations on T_50_ are limited by the absence of a healthy control group, and alterations of T_50_ in PA compared to healthy conditions require further study. We cannot rule out other determinants influencing the serum calcification propensity in RH patients, which are not primarily mediated by elevated aldosterone concentration. In patients with arterial hypertension, lower T_50_-values were observed as compared to healthy controls (33). In our cohort, no difference in T_50_-values were detected between the PA and RH groups, although RH patients had a higher cardiovascular risk score. Based on these observations, it could be speculated that higher cardiovascular risk would be translated into lower T_50_-values. In theory, as these T_50_-values did not differ between PA and RH patients, these results could indirectly suggest an additional effect of aldosterone on calcification propensity as a novel independent non-traditional cardiovascular risk factor (23). Indeed, in patients with PA, the ARR was inversely related to endogenous calcification inhibitory mechanisms, as high ARRs were related to lower T_50_-values.

According to results from the general population and patients with impaired renal function, lower T_50_-values are consistently related to higher cardiovascular mortality (23, 25, 26). Due to the small population and the short follow-up, we did not analyze cardiovascular mortality. Instead, we addressed the cardiovascular morbidity risk by using the well-established ACSVD score which showed a significant correlation between high ACSVD score and low T_50_-values, especially in patients with PA. Long term follow up studies are in need to confirm the increased cardiovascular risk related to low T_50_-values. In addition to the ACSVD score, other laboratory parameters which are traditionally related to higher cardiovascular morbidity such as high phosphate and lower HDL concentration were also significantly associated with lower T_50_-values (23, 25, 26, 33).

In order to assess the clinical impact of the T_50_ test in patients with PA and to determine its relationship with aldosterone and vascular calcification, we analyzed the available CT scans from patients with PA which were performed at the time of diagnosis. We found that T_50_-values correlated with the extent of vascular calcification of the aorta. Patients with low T_50_-values showed increased equivalent calcification mass and higher abdominal Agatston score. Previous studies in patients with chronic kidney disease have shown an association of T_50_ with coronary calcification progression (34) and aortic stiffness (24) therefore the present study provides additional information on clinical impact of low T_50_-values among these patients. Various clinical and experimental studies highlighted the important role of aldosterone in the development of vascular calcification (13, 35, 36). High aldosterone concentrations are associated with subclinical coronary atherosclerosis and mortality in general population (37) and in patients with PA an early development of vascular calcification is observed (18). The use of MRA on the other hand may ameliorate vascular calcification (3, 38). According to experimental studies, aldosterone exerts its effects at least partly by binding to the mineralocorticoid-receptor in vascular smooth muscle cells (39). Multiple experimental studies show that elevated aldosterone concentration promotes the transition of vascular smooth muscle cells toward an osteogenic phenotype and thereby promotes calcification (40). However, the current observations do not provide clear evidence that these effects of aldosterone may be reflected in a worsened serum calcification propensity of PA patients as compared to RH patients. A further comparison with healthy patients would be necessary to clearly differentiate alterations of T_50_-time in these collectives. Furthermore, in the present study, treatment of PA patients either by adrenalectomy or by medical treatment with MR antagonists did not increase but rather decrease T_50_, while a recent study performed in hemodialysis patients has shown a slight increase of T_50_ under spironolactone treatment (41). Hypothetically, other systemic and renal effects of PA might indirectly compensate for more direct effects of aldosterone on calcification propensity, which could be relevant for the discrepancy between MRA effects in these populations. The different level of MR activation and therapeutic inhibition, as well as mean aldosterone concentrations or prolonged effects of aldosterone in PA patients in the absence of chronic kidney disease might play a role on calcification propensity. In addition, aldosterone-independent activation of the MR and modulation of its activity may be especially relevant in uremic conditions (42, 43).

Most studies on T_50_ were based on the evaluation of T_50_ at a single time point, and less information on longitudinal alterations of T_50_ are available. Recent observations showed that the decrease in T_50_ from 246 ± 64 to 190 ± 68 min in 2 years among hemodialysis patients was an independent risk factor for mortality in this cohort (44). On the other hand, longer maturation of CPPs or higher T_50_ would translate into improved calcification propensity. As described above, due to short follow up we were not able to evaluate the mortality risk. Since prolongation of T_50_-time might be a therapeutic target, we aimed to identify factors determining T_50_ change over time in our population. First, in PA patients, higher calcium concentration at study entry was related to decrease in T_50_ during the follow up period. Although calcium concentration stayed within normal range, it had a significant negative effect on calcification propensity. The relationship between aldosterone and calcium is complex. High aldosterone levels induce calciuria by activating calcium transport in renal tubuli. This effect causes hypocalcemia which further activates PTH secretion to compensate this mechanism (45–48). Moreover, higher normal calcium concentration could be explained by chronic vitamin D supplementation. Unfortunately, we were not able to rule out the effect of PTH concentration or vitamin D supplementation on calcium levels and T_50_ in these patients. Secondly, a decrease in magnesium concentration as low as −0.3 ± 0.3 mmol/l had a significant effect on T_50_ reduction in patients with PA. Magnesium counteracts the osteogenic effects of calcium-phosphate on vascular smooth muscle cells and reduces vascular smooth muscle cell calcification (49, 50). There is growing evidence indicating that low normal magnesium concentration or lower dietary intake are associated with higher cardiovascular risk (51–54). Magnesium supplementation in turn is associated with beneficial effects on serum calcification propensity. An increment of 0.2 mmol/l was able to prolong T_50_ for 51 ± 15 min for healthy people and 44 ± 13 min for dialysis patients (55, 56). The effect of aldosterone on magnesium handling is still incompletely understood (57). Since aldosterone may induce magnesuria, the mechanisms underlying the observed associations in this cohort of PA patients appear elusive (57). Thirdly, in accordance with previous observations (58), we have observed that an increase in phosphate levels over the time was related to a lowering of T_50_. In addition, we have observed that patients with impaired renal function at baseline tended to have a decrease in T_50_ over the time, however a decrease in renal function during the follow up period did not significantly affect calcification propensity. This may be explained by the relatively short observation period. Taken together, longitudinal evaluation suggests, that T_50_ is at least partially determined by calcium, phosphate and magnesium in PA-patients, which are known to contribute to T_50_ (22). To which extent altered mineral homeostasis due to renal and extrarenal effects of PA underly T_50_ in these patients requires further study. Although the current observations cannot rule out the involvement of other factors, control of these parameters could be a target for future study in order to prevent predisposition to calcification and cardiovascular risk in PA.

The present study has several strengths and limitations. Although we present T_50_ results from a cohort which is quite susceptible to cardiovascular mortality, our study population is small, with unequal sample size in the groups, observational and lacks a healthy control group, which might increase the possibility of biased associations. Furthermore, effects could be masked by scatter of measurements in such a small sample size. Due to short follow up we were not able to assess the cardiovascular mortality risk of these patients. On the other hand, the strength of the study is that it was performed in one center ensuring a strict work up and follow-up schedule, as well as strict and uniform pre-analytical sample processing. We have included vascular calcification measurements enabling the analysis of direct association of T_50_ with clinical outcome and intra-individual changes of T_50_ with regard to treatment.

To conclude, although higher aldosterone-renin-ratio was related to lower T_50_-values, normalization of ARR either by the surgery or medication was not associated with increased T_50_ in patients with PA, but rather a lower T_50_ was observed at follow-up. Shortening of T_50_ over the observational period was associated with higher calcium levels, an increase in phosphate levels or decrease in magnesium concentrations in PA patients. Most importantly, serum calcification propensity was associated with ACSVD cardiovascular risk score and aortic calcification in PA patients. Further studies are warranted to confirm the current observations and whether therapies targeted to increase T_50_-time might benefit patients with PA.

## Data Availability Statement

The raw data supporting the conclusions of this article will be made available by the authors, without undue reservation.

## Ethics Statement

The study was approved by the local Ethics Committee of the medical faculty of the Heinrich-Heine-University Düsseldorf (Study numbers: 3848 and 3919) and carried out in accordance with the Declaration of Helsinki. The patients/participants provided their written informed consent to participate in this study.

## Author Contributions

LS, LR, JV, and JS: conceptualization. AP, MK, KB, and MB: formal analysis. TK, CS, KB, and MK: investigation. MK, LR, JV, and JS: writing—original draft preparation. TK, KE, LS, AP, LR, JV, and JS: writing—review and editing. JV and JS: supervision. All authors contributed to the article and approved the submitted version.

## Funding

This work was supported by Forschungskommission of the Medical Faculty, Heinrich-Heine-Universität Düsseldorf (KS9772730) and the Deutsche Forschungsgemeinschaft (VO2259/2-1).

## Conflict of Interest

AP is an employee and stockholder of Calciscon AG, which commercializes the T50 test. The remaining authors declare that the research was conducted in the absence of any commercial or financial relationships that could be construed as a potential conflict of interest.

## Publisher's Note

All claims expressed in this article are solely those of the authors and do not necessarily represent those of their affiliated organizations, or those of the publisher, the editors and the reviewers. Any product that may be evaluated in this article, or claim that may be made by its manufacturer, is not guaranteed or endorsed by the publisher.
