# Supplementary material for: Serum Calcification Propensity and Calcification of the Abdominal Aorta in Patients With Primary Aldosteronism
Source: Front Cardiovasc Med. 2022 Jan 24;9:771096. doi: 10.3389/fcvm.2022.771096 (PMC8818752; doi:10.3389/fcvm.2022.771096)
Supplement: Supplementary file 1 [file Data_Sheet_1.pdf]

Supplementary Material

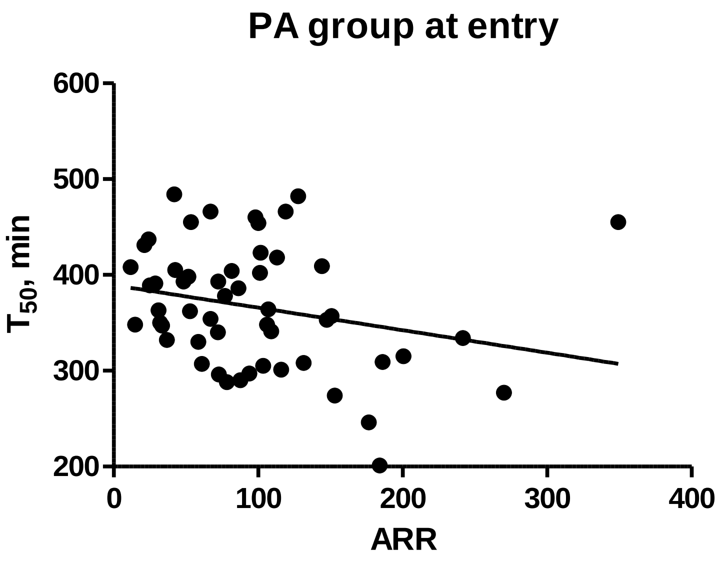

Figure 1. Relationship between calcification propensity and aldosterone renin ration (ARR) at study entry among patients diagnosed with PA. T_{50} represents the half maximal transformation time of primary calciprotein particles to secondary ones and is expressed in minutes. At study entry a negative relationship between T_{50} and ARR (r -0.282, $p < 0.05$) was observed.

Variable	All patients		PA		RH	
	β	p	β	p	β	p
Phosphate	- 0.320	**0.031**			- 0.170	0.297
HDL	0.012	0.938			0.605	**0.001**
ACSVD	- 0.252	0.160	- 0.134	0.397		
ARR			- 0.365	**0.025**	0.355	**0.019**

Table 1. Multivariate linear regression model assessing the relationship between calcification propensity (T_{50}) measured at the study entry and various variables. PA – primary aldosteronism, RH – resistant hypertension. HDL – high density lipoprotein, ACSVD - Atherosclerotic cardiovascular disease score, ARR – aldosterone renin ration, β – correlation coefficient. Variables which showed a significant relationship with calcification propensity (T_{50}) in univariate regression analysis were included into multivariate analysis. Significance with $p < 0.05$

	PA		RH	
	At entry	Follow up	At entry	Follow up
Number of patients	66	22	28	28
M:F	1.8:1	1:1	1.8:1	1.8:1
Age, y	53.5 ± 12.4	53.0 ± 10.1	58.0 ± 11.2	59.1± 12.3
SBP, mmHg	154 ± 11	128 ± 19*	156 ± 21	150 ± 25
DBP, mmHg	88 ± 8	77 ± 10*	91 ± 10	89 ± 12
ACSVD score, %	9.6 (4.7 – 16.8)	12.2 ± 9.9	24.9 (17.5 – 30)	20.1 ± 9.0
Aldosterone, ng/L	187.3 (119 – 241)	120 (66 – 304)	99 (42 – 148)	91 (63 – 133)
Renin, pg/ml	1.9 (1.2 – 2.8)	15 (7.7 – 45)***	11 (4.4 – 59)	16.0 (5.1 – 69)
ARR	85.3 (52.4 – 134.4)	8.7 (2.5 – 30.3)***	4.6 (0.9 – 22.9)	4 (0.9 – 15.4)
Potassium mmol/L	3.6 ± 0.6	4.4 ± 0.3 ***	4.1 ± 0.6	4.1 ± 0.6
Creatinine, mg/dl	0.9 (0.8 – 1.1)	1 (0.9 – 1.1)***	0.9 (0.8 – 1.3)	1 (0.8 – 1.5)
eGFR, ml/min/1.73m^2	82 (72.7 – 95.5)	74 (61 – 85) ***	74.5 (54.5 – 96.5)	75.5 (45 - 90)*
Calcium, mmol/L	2.33 ± 0.1	2.37 ± 0.1*	2.30 ± 0.1	2.34 ± 0.1
Phosphate, mmol/L	0.97 ± 0.3	1.01 ± 0.2	1.01 ± 0.2	1.19 ± 0.3**
Magnesium, mmol/L	0.83 ± 0.1	0.81 ± 0.1	0.86 ± 0.1	0.86 ± 0.1
Triglyceride, mg/dl	127 (96 – 183)	166 (120 – 207)**	119 (93 – 206)	125 (105 – 183)
HDL, mg/dl	48 (41 – 61)	52 (41 – 69)	45 (37 – 57)	44 (37 – 55)
Albumin, g/dl	4.4 ± 0.3	4.5 ± 0.3	4.4 ± 0.5	4.6 ± 0.5
CRP, mg/dl	0.3 (0.2 – 0.5)	0.3 (0.1 – 0.4)	0.4 (0.3 – 1.1)	0.3 (0.2 – 0.5)
$HbA1_C$, %	5.4 (5.1 – 5.8)	5.6 (5.3 – 6)*	5.8 (5.6 – 6.4)	6.0 (5.5 – 6.5)
T_{50}, min	371 ± 65	354 ± 52*	382 ± 44	367 ± 56

Table 2. Characteristics of study population at entry and follow up visit. PA – primary aldosteronism, RH – resistant hypertension, SBP – systolic blood pressure, DBP – diastolic blood pressure, ACSVD - Atherosclerotic cardiovascular disease score, ARR – aldosterone renin ratio, eGFR – estimated glomerular filtration rate, HDL – high density lipoprotein, CRP – C – reactive protein, HbA1C – glycosylated hemoglobin, T_{50} – calcification propensity score. Dichotomous data are presented as percentages whereas continuous data as means ± SD or median (Q1 – Q3). *** represent significant difference between entry and follow up within the group with $p < 0.001$, ** $p < 0.01$, * $p < 0.05$ using paired t-test or Wilcoxon signed rank test.
